# Highly predictive SNP markers for efficient selection of the wheat leaf rust resistance gene *Lr16*

**DOI:** 10.1186/s12870-017-0993-7

**Published:** 2017-02-15

**Authors:** Mulualem T. Kassa, Frank M. You, Colin W. Hiebert, Curtis J. Pozniak, Pierre R. Fobert, Andrew G. Sharpe, James G. Menzies, D. Gavin Humphreys, Nicole Rezac Harrison, John P. Fellers, Brent D. McCallum, Curt A. McCartney

**Affiliations:** 1 Agriculture and Agri-Food Canada, Morden Research and Development Centre, 101 Route 100, Morden, MB R6M 1Y5 Canada; 2 National Research Council, 110 Gymnasium Place, Saskatoon, SK S7N 0W9 Canada; 3 University of Saskatchewan, Crop Development Centre, 51 Campus Drive, Saskatoon, SK S7N 5A8 Canada; 4 University of Saskatchewan, Global Institute for Food Security, 110 Gymnasium Place, Saskatoon, SK S7N 4J8 Canada; 5 Agriculture and Agri-Food Canada, Ottawa Research and Development Centre, 1341 Baseline Road, Ottawa, ON K1A 0C5 Canada; 6 Department of Plant Pathology, Kansas State University, Manhattan, KS 66506 USA; 7 USDA–ARS, Hard Winter Wheat Genetics Research Unit, Manhattan, KS 66506 USA

**Keywords:** *Lr16*, Leaf rust, *Puccinia triticina*, Wheat, *Triticum aestivum* L, Disease resistance, Single nucleotide polymorphism (SNP), Linkage analysis, Resistance gene analog (RGA)

## Abstract

**Background:**

*Lr16* is a widely deployed leaf rust resistance gene in wheat (*Triticum aestivum* L.) that is highly effective against the North American *Puccinia triticina* population when pyramided with the gene *Lr34. Lr16* is a seedling leaf rust resistance gene conditioning an incompatible interaction with a distinct necrotic ring surrounding the uredinium. *Lr16* was previously mapped to the telomeric region of the short arm of wheat chromosome 2B. The goals of this study were to develop numerous single nucleotide polymorphism (SNP) markers for the *Lr16* region and identify diagnostic gene-specific SNP marker assays for marker-assisted selection (MAS).

**Results:**

Forty-three SNP markers were developed and mapped on chromosome 2BS tightly linked with the resistance gene *Lr16* across four mapping populations representing a total of 1528 gametes. Kompetitive Allele Specific PCR (KASP) assays were designed for all identified SNPs. Resistance gene analogs (RGAs) linked with the *Lr16* locus were identified and RGA-based SNP markers were developed. The diagnostic potential of the SNPs co-segregating with *Lr16* was evaluated in a diverse set of 133 cultivars and breeding lines. Six SNP markers were consistent with the *Lr16* phenotype and are accurately predictive of *Lr16* for all wheat lines/cultivars in the panel.

**Conclusions:**

*Lr16* was mapped relative to SNP markers in four populations. Six SNP markers exhibited high quality clustering in the KASP assay and are suitable for MAS of *Lr16* in wheat breeding programs.

**Electronic supplementary material:**

The online version of this article (doi:10.1186/s12870-017-0993-7) contains supplementary material, which is available to authorized users.

## Background

Wheat (*Triticum aestivum* L.) is an important food crop providing a fifth of the world’s calorie intake. Wheat is grown on more than 215 million hectares and cultivated across more regions of the world than any other staple crop [1]. Wheat diseases caused by various pathogens cause significant yield loss across the world. Of these, the rust fungi cause serious diseases of wheat and pose significant constraints to wheat production. The three wheat rust diseases are stem, leaf, and stripe (or yellow) rust, caused by *Puccinia graminis* f. sp. *tritici* Eriks. & E. Henn., *Puccinia triticina* Eriks., and *Puccinia striiformis* Westend. f.sp. *tritici* Erikss., respectively [2].

Wheat leaf rust occurs more frequently and is more prevalent globally than other cereal rust pathogens [3, 4]. In the eastern prairies of Canada, annual yield losses due to leaf rust are estimated at 5 to 15% when susceptible wheat cultivars are grown [4]. Leaf rust can be effectively controlled by applying fungicides or through genetic resistance. Of these, host resistance is the most efficient, economical, and environmentally effective means to control this disease. In addition, there is the possibility that rust pathogens may develop resistance to fungicides [5]. To date, 73 numerically designated wheat leaf rust resistance (*Lr*) genes have been identified and catalogued [6–8]. Additionally, a small number of race-specific seedling stage leaf rust resistance genes have been cloned, including *Lr1*, *Lr10*, and *Lr21* [9–11]. All of these resistance (R) genes were found to belong to the coiled-coil, nucleotide binding site, leucine rich repeat (CC-NB-LRR) class of R proteins. *Lr34* was the first wheat resistance gene to be cloned that provides partial resistance to multiple pathogens [12]. More recently, the partial leaf rust resistance gene *Lr67* was also cloned [13]. *Lr34* and *Lr67* encode a putative ABC transporter and a hexose transporter, respectively. *Lr16* is likely a member of the CC-NB-LRR class of R proteins given its activity at the seedling stage and race-specificity. Although many wheat leaf rust (*Lr*) resistance genes have been identified, virulence mutations in the *P. triticina* population have overcome host resistance conferred by a number of *Lr* genes (i.e., race-specific R genes). To mitigate the problem posed by the evolution of new virulent races of the pathogen, deployment of multiple *Lr* genes or gene pyramiding is recommended to promote the durability of *Lr* genes [2].

*Lr16* is a seedling leaf rust resistance gene, conditioning an incompatible interaction with a distinct necrotic ring surrounding the uredinium [14]. In Canada, *Lr16* was first deployed in the variety Selkirk [4]. Virulence to *Lr16* was first detected in Canada in 1961. Although considered a defeated gene, *Lr16* still retains a partial resistance effect against virulent *P. triticina* isolates and still offers protection against wheat leaf rust in Canada [14, 15]. It is widely deployed and is particularly effective against the North American *P. triticina* population when pyramided with *Lr34* [16–18]. The exact nature of the interaction with *Lr34* is not understood, but similar interactions were reported between *Lr34* and *Lr1*, *Lr2a*, *Lr3*, *Lr3ka*, *Lr11*, *Lr13*, *Lr17*, and *LrB* [16]. Genetic analysis has revealed the presence of *Lr16* in the Canadian wheat cultivars ‘AC Domain’, ‘AC Karma’, ‘AC Majestic’, ‘AC Splendor’, ‘Columbus’, the American cultivar ‘Grandin’, and in other cultivars elsewhere [14, 19]. Resistance conferred by *Lr16* has been utilized in wheat breeding programs in Canada and around the world.

*Lr16* has been mapped to the terminal region of wheat chromosome arm 2BS [14, 20]. Previous studies have identified simple sequence repeat (SSR) markers that were linked with *Lr16* on chromosome arm 2BS [14, 20]. However, lack of distally flanking markers has hampered the fine mapping and cloning efforts of *Lr16* and slowed the development of diagnostic molecular markers for marker-assisted selection (MAS). Thus, the main objectives of the present study were to map *Lr16* in multiple mapping populations, develop numerous SNP markers for the *Lr16* region, and identify SNP markers that are tightly linked with *Lr16* and useful for efficient selection of *Lr16* in wheat breeding programs.

## Methods

### Mapping populations

Four mapping populations were used in this study. An F_6_‐derived recombinant inbred line (RIL) population (*n* = 384) was developed from the cross of BW278 (‘AC Domain’*2/‘Sumai 3’) with ‘AC Foremost’ (HY320*5/BW553//HY320*6/7424-BW5B4; [21]). BW278 is a Canadian spring wheat breeding line that carries *Lr16* (resistant parent) and ‘AC Foremost’ is Canadian spring wheat cultivar that is susceptible to *P. triticina* isolate 12–3 MBDS at the seedling stage. The ‘AC Majestic’/‘Glenlea’ population consisted of 400 F_1_-derived doubled haploid (DH) lines. ‘AC Majestic’ (‘Columbus’*2//‘Saric 70’/‘Neepawa’/3/‘Columbus’*5//‘Saric70’/‘Neepawa’) is a Canadian spring wheat cultivar that carries *Lr16* (resistant parent) while ‘Glenlea’ (UM530/CB100; [22]) is a Canadian spring wheat cultivar that is susceptible to *P. triticina* isolate 12–3 MBDS at the seedling stage. The RL4452/‘AC Domain’ F_1_-derived doubled haploid population (*n* = 172) was generated from the cross of the susceptible wheat line RL4452 (‘Glenlea’*6/‘Kitt’), which is susceptible to *P. triticina* isolate 12–3 MBDS at the seedling stage, with the resistant (*Lr16* carrier) Canadian spring wheat ‘AC Domain’ (ND499/RL4137//ND585; [23]). The fourth population was developed from a RIL population of 94 lines of the cross ‘Kenyon’/86ISMN 2137. ‘Kenyon’ (‘Neepawa’*5/‘Buck Manantial’; [24]) is a Canada Western Red Spring (CWRS) cultivar that carries *Lr16* (resistant), while 86ISMN 2137 is of unknown origin and is susceptible to *P. triticina* isolate 12–3 MBDS at the seedling stage. The ‘Kenyon’/86ISMN 2137 mapping population was developed and provided by Dr. G.R. Hughes, University of Saskatchewan, Crop Development Centre. All other mapping populations were developed by the authors at Agriculture and Agri-Food Canada.

### Seedling tests with race MBDS (12–3)

Tests for leaf rust resistance conditioned by *Lr16* were done at the seedling stage as previously described [25]. Seeds were planted in clumps of approximately 10 seeds evenly spaced in fibre flats (25 × 15 cm). Approximately 14 d after seeding, the seedlings at the two leaf stage were inoculated with urediniospores of *P. triticina* isolate 12–3 MBDS (nomenclature as previously described [26]) mixed with a light mineral oil (Bayol, Esso Canada, Toronto, ON) sprayed onto the leaves using a compressed air sprayer. This *P. triticina* isolate is fully avirulent on *Lr16* carriers. The plants were allowed to dry, to evaporate the mineral oil, for at least 1 h then moved to a 100% humidity cabinet for approximately 17 h of incubation. The plants were then moved to a greenhouse at 20 ± 4 °C with supplemental lighting. After approximately 14 d, plants were rated for symptoms using a ‘0’ to ‘4’ infection scale where ‘0’ (no symptoms), ‘;’ (hypersensitive flecks), ‘1’ (small uredinia with necrosis), and ‘2’ (small to medium-sized uredinia with chlorosis) were considered resistant responses and ‘3’ (medium-sized uredinia without necrosis or chlorosis) and ‘4’ (large-sized uredinia without necrosis or chlorosis) were considered susceptible responses [26].

### DNA marker development

SSR markers linked to *Lr16* were identified for analysis based on previous research [14]. Previous studies mapped *Lr16* to the terminal region of wheat chromosome bin 2BS3 [fraction length: 0.84–1.00]. All sequences of previously mapped SSR, Diversity Arrays Technology (DArT), and SNP markers near *Lr16* were used as queries in the BLASTN search of the *Brachypodium* genome to identify orthologous loci. Wheat EST (wEST) that were mapped to bin 2BS3 (FL 0.84-1.00) and publically available wEST sequences which are orthologous to putative *Brachypodium* genes within the syntenic genomic region on Chromosome 5 were used for developing conserved primers using ConservedPrimers 2.0 software [27].

SNP markers were developed with multiple strategies. Linked wheat 90 K Infinium SNP markers [28] were identified by genotyping the RL4452/‘AC Domain’ and BW278/‘AC Foremost’ mapping populations. KASP assays were designed for these SNPs and tested on the other populations.

In addition, a BLAST search using sequences of previously mapped markers on chromosome arm 2BS was performed to identify putative SNP markers linked to the *Lr16* locus. The wheat 90 K iSelect Infinium assay [28] and the SNP database at CerealsDB [29] were used to mine SNPs. Additional SNP markers were also developed through comparative synteny analysis using *Brachypodium* and rice genomes. Here, syntenic *Brachypodium* and rice genes were used as queries in BLASTN searches against the wheat chromosome arm 2BS survey sequence to identify putative syntenic wheat genes. These wheat genes were then used as queries in BLASTN searches of the CerealsDB SNP database [29]. Genomic resources at NCBI [30] and GrainGenes [31] were utilized to discover additional SNP markers linked with *Lr16*.

Whole exome capture (WEC) data [32] of chromosome 2BS was also utilized to mine SNP markers associated with *Lr16*. Bulked segregant analysis (BSA) coupled with WEC sequencing was used to identify SNPs. Four sets of DNA (BW278 [*Lr16*-carrier], bulk of 15 *Lr16*-carrier BW278/‘AC Foremost’ RILs, ‘AC Foremost’ [non-carrier], and bulk of 15 susceptible BW278/‘AC Foremost’ RILs) were sequenced with Illumina short read technology (2 × 100 bp) and assembled against the WEC reference sequence. SNP variants were called that accurately differentiated the resistant and susceptible lines and bulks, respectively.

Finally, all potential genes from chromosome arm 2BS survey sequence of hexaploid wheat variety Chinese Spring [33] were predicted using gene prediction software GeneMark [34]. Resistance gene analogs (RGAs) from the genes on 2BS were identified using the RGA prediction pipeline program RGAugury [35], although Chinese Spring does not have *Lr16*. RGAs linked with the *Lr16* locus were detected using previously mapped markers. BLAST was used to identify wheat RGAs co-linear with RGAs in *Brachypodium* and rice as described above. RGA-based SNP markers were developed from sequences of RGAs Sanger sequenced in wheat lines with and without *Lr16*. Moreover, a BLAST search of SNPs from the CerealsDB wheat SNP database [29] and the 90 K wheat Infinium SNP array was conducted using sequences of the filtered RGA contigs as queries to identify SNP markers putatively linked to the *Lr16* locus. RGA-based markers linked with *Lr16* were also identified from RNA sequences (RNA-seq) and an *in silico* subtraction method [36]. Polymorphic SNPs were genotyped on the appropriate mapping populations and used for linkage analysis.

### DNA marker analyses and genotyping

Genomic DNA for the populations, ‘AC Majestic’/‘Glenlea’, RL4452/‘AC Domain’ and Kenyon/86ISMN 2137, were extracted from lyophilized fresh leaf tissue using the DNeasy Plant DNA extraction kit (Qiagen, Toronto, Canada). For the BW278/‘AC Foremost’ population, DNA from lyophilized fresh leaf tissue was extracted using a modified ammonium acetate method as described previously [37]. Stock DNA concentration was estimated with a fluorometer using Hoechst 33258 stain and diluted to a working concentration of 15 ng/μl. All of the SNPs identified were genotyped using the KASP assay [38]. Using DNA sequence flanking the variant SNP position, two allele-specific forward primers and one common reverse primer were designed (Additional file 1: Table S1). PCR conditions and KASP assays were performed using methods as previously described [37]. Fluorescence detection of the PCR products was performed using an Omega Fluorostar plate reader (BMG LABTECH GmbH, Ortenberg, Germany). The data were analyzed using KlusterCaller software (LGC Genomics, Beverly, USA). Only SNP markers that showed high quality allele calls were used for linkage analysis. The quality of the marker was determined based on the cluster quality of the scatter plot and by comparing the allele call of each genotype with alleles of the parental lines. Datapoints that did not fit within clusters were scored as missing data and were excluded from linkage analysis. PCR conditions and genotyping methods for SSR markers were previously described [14], and the same protocol was used to test the sequence characterized amplified region (SCAR) marker pwm6 and the ConservedPrimer marker pwm16.

### Linkage analysis

A linkage map of the region of chromosome arm 2BS carrying *Lr16* was constructed for each mapping population using MapDisto 1.7.7 software [39]. Loci were analyzed for conformation to a Mendelian segregation ratio (1:1) using a *χ* ^2^ test. A minimum LOD (logarithm of odds) threshold of 3.0 and maximum recombination fraction of 0.3 were used to identify linkage groups. Recombination fractions were converted into map distances using the Kosambi mapping function [40].

### Haplotype analysis of wheat lines with *Lr16* SNP markers

Marker haplotype analysis was performed on a panel of 133 wheat lines and cultivars to evaluate the diagnostic potential of SNP markers linked with *Lr16* for MAS. This collection of wheat germplasm was assembled by Dr. D.J. Somers, formerly of Agriculture and Agri-Food Canada, from western Canadian wheat breeders. The wheat cultivars and breeding lines were previously tested with *P. triticina* isolate 12–3 MBDS and have known infection types. The panel consists of cultivars and breeding lines from nine Canadian wheat marketing classes, which represent different grain quality profiles for different end-uses. Considerable diversity exists between these marketing classes. The haplotype panel also included wheats from 10 additional countries to further broaden the diversity sampled (Table 2 and Additional file 1: Table S2). Most wheat lines in the haplotype panel were susceptible to isolate 12–3 MBDS (i.e., do not carry *Lr16*). The few resistant lines included in the haplotype panel were known carriers of *Lr16*.

## Results

### Phenotypic evaluation

Each of the mapping populations (BW278/‘AC Foremost’ RIL, ‘Majestic’/‘Glenlea’ DH, RL4452/‘AC Domain’ DH, and ‘Kenyon’/86ISMN2137 RIL) fitted a 1 resistant : 1 susceptible segregation ratio, indicating single gene segregation for resistance to *P. triticina* isolate 12–3 MBDS (Table 1). Although each of these parental lines carries a number of leaf rust resistance genes, the isolate used here (12–3 MBDS) was virulent on those genes, except for *Lr16* on which it was avirulent. Resistant lines in these populations had the infection type ‘1’ with a large necrotic ring around a small uredinium, characteristic of *Lr16*, whereas susceptible lines had infection types ‘3’ or ‘4’.

**Table 1:** Segregation of *Lr16* in four wheat populations, including *χ* ^2^ for fit to a 1:1 ratio and corresponding probability

Population	Resistant	Susceptible	Total	χ^2^_1:1_	P
BW278/‘AC Foremost’	187	197	384	0.260	0.610
‘AC Majestic’/‘Glenlea’	196	204	400	0.160	0.689
RL4452/‘AC Domain’	91	80	171	0.708	0.400
‘Kenyon’/86ISMN 2137	42	45	87	0.103	0.748

### Marker development

The SCAR marker pwm6 was developed from the DArT marker wPt-5960 that mapped near *Lr16*, and the ConservedPrimer marker pwm16 was developed from the wEST BF483211 that was mapped on the distal end of chromosome 2BS.

SNP markers were identified from publicly available wheat genomic resources and SNP databases using sequences of previously mapped markers linked to the *Lr16* locus on chromosome 2BS, syntenic genes of *Brachypodium* and rice genomes and from RGAs identified from RNA-seq and *in silico* subtraction. SNPs were also identified from sequences of RGAs that were tightly linked with *Lr16*. A total of 219 putative SNP loci were identified. Of these, 83 SNPs were mined from CerealsDB using wESTs, SSRs, and DArTs located on chromosome arm 2BS as BLAST queries or were reported on CerealsDB as tentatively mapped to chromosome arm 2BS [29], 35 SNPs from 90 K iSelect Infinium Array [28], six SNPs were from RGAs identified through RNA-seq and *in silico* subtraction analysis [36], 64 SNPs were from the exome sequence of BW278/‘AC Foremost’ population [32], and 21 SNPs were identified from CerealsDB or the 90 K wheat Infinium SNP array using syntenic genes in *Brachypodium* and rice genomes as BLAST queries. Additionally, 10 SNP markers were derived from coding sequence (CDSs) of RGAs tightly linked with *Lr16*. KASP assays were designed for SNPs and tested on the parents of the four mapping populations, which yielded 70 (32%) polymorphic SNPs that were tested for linkage.

### *Lr16* genetic linkage map

**Fig. 1:**
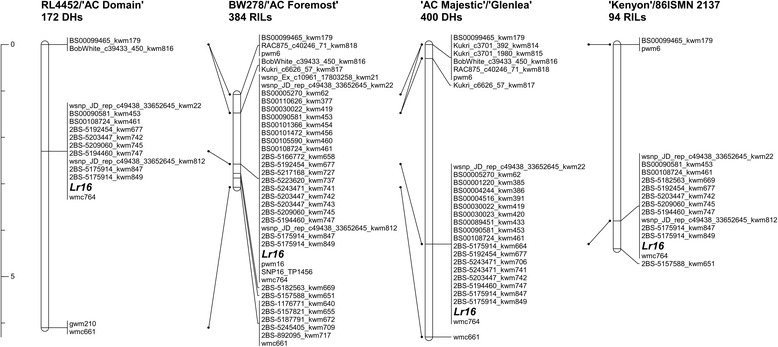
Genetic linkage maps calculated for four mapping populations showing the location of the leaf rust resistance gene *Lr16* on wheat chromosome arm 2BS. *Lines* identify markers in common between adjacent linkage maps. The ruler on the *left* of the linkage maps is scaled in centiMorgans (cM)

A total of 43 high quality new SNP markers and two PCR markers (SCAR pwm6 and ConservedPrimer marker pwm16) were mapped on chromosome arm 2BS linked with the resistance gene *Lr16* across the four mapping populations (Fig. 1; Additional file 1: Table S2). The SNPs were selected based on cluster quality of the allele calls as revealed on the KASP genotyping assay. Three SSR markers (wmc764, gwm210 and wmc661) were previously mapped relative to *Lr16* [14]. Genetic positions and marker order were consistent across the four populations (Fig. 1) and no marker deviated from the expected 1:1 Mendelian segregation ratio (data not shown). In all four populations, *Lr16* was flanked distally by the SNP marker BS00099465_kwm179 while the SSR marker wmc661 was the proximal flanking marker in all populations except ‘Kenyon’/86ISMN 2137. The SNP marker 2BS-5157588_kwm651 flanked *Lr16* proximally in both the ‘Kenyon’/86ISMN 2137 and BW278/‘AC Foremost’ populations. It is worth noting that 2BS-5182563_kwm669 was the closest proximal flanking marker of *Lr16* in BW278/‘AC Foremost’ while it co-segregated with *Lr16* in ‘Kenyon’/86ISMN 2137. This result was not unexpected given the higher genetic resolution of BW278/‘AC Foremost’, which consisted of 384 RILs (i.e., approximately equivalent to 768 gametes). Similarly, the SNP marker Kukri_c6626_57_kwm817 was the closest distal flanking marker in the BW278/‘AC Foremost’ and ‘AC Majestic’/‘Glenlea’ populations. One SSR and nine SNP markers were mapped in all the four populations while the remaining markers were mapped in one, two or three populations (Fig. 1). Of the ten markers mapped in all populations, an SSR (wmc764) and eight SNP markers (wsnp_JD_rep_c49438_33652645_kwm22, BS00090581_kwm453, BS00108724_kwm461, 2BS-5192454_kwm677, 2BS-5203447_kwm742, 2BS-5194460_kwm747, 2BS-5175914_kwm847, and 2BS-5175914_kwm849) co-segregated with *Lr16*. Five of the co-segregating SNPs were derived from three RGAs found in the *Lr16* region. The SNPs BS00108724_kwm461, 2BS-5175914_kwm847, and 2BS-5175914_kwm849 were derived from the same RGA, while 2BS-5203447_kwm742 and 2BS-5194460_kwm747 were from two additional RGAs. A SNP marker identified from RNA-seq and *in silico* subtraction analysis (SNP16_TP1456) co-segregated with *Lr16* in the BW278/‘AC Foremost’ population. SNP16_TP1456 and 2BS-5194460_kwm747 were derived from the same RGA.

### Haplotype analysis

**Fig. 2:**
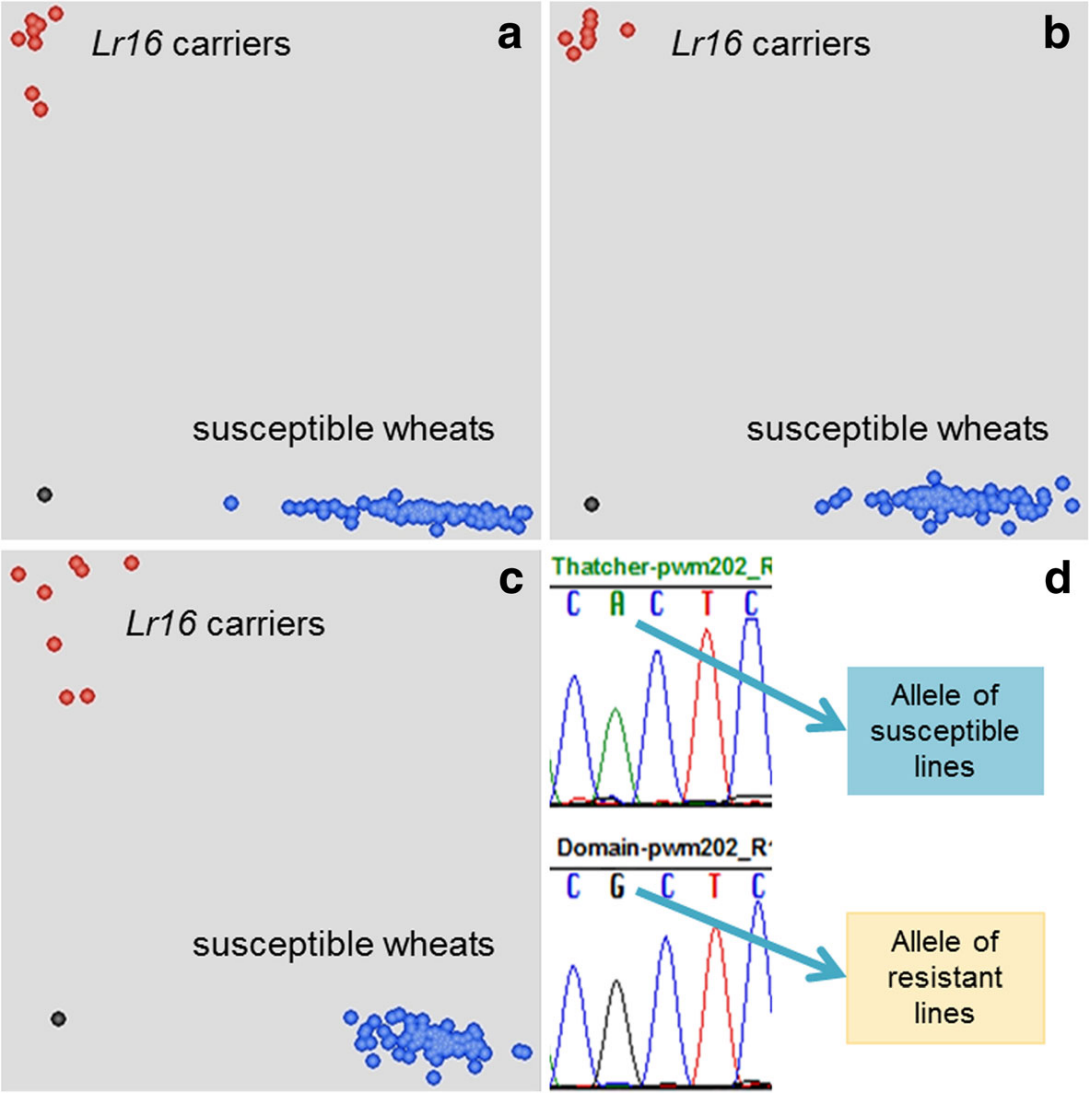
Cartesian cluster plots of KASP markers (**a**) 2BS-5175914_kwm849, (**b**) 2BS-5192454_kwm677, (**c**) 2BS-5175914_kwm847 tested on a set of 133 wheat lines to show the diagnostic potential of SNP markers for high-throughput MAS. The x-axis and y-axis are the fluorescence intensities of FAM and HEX, respectively. *Black* datapoints are no template controls. **d** Sanger sequencing chromatograms, for ‘Thatcher’ and ‘AC Domain’, of an RGA co-segregating with *Lr16* from which the SNP marker 2BS-5175914_kwm849 was identified

The eight SNP markers that co-segregated with *Lr16* in all four populations were tested on a diverse set of 133 wheat lines and cultivars (Additional file 1: Table S2). All lines and cultivars were previously tested with leaf rust race 12–3 MBDS and have a known genotype at the *Lr16* locus. The panel was comprised of 10 resistant lines (*Lr16*+), while the remaining lines were susceptible (*Lr16*-) (Table 2). Six of the SNP markers (BS00108724_kwm461, 2BS-5192454_kwm677, 2BS-5203447_kwm742, 2BS-5194460_kwm747, 2BS-5175914_kwm847, and 2BS-5175914_kwm849) were consistent with *Lr16* phenotype and were completely predictive of *Lr16* for all wheat lines/cultivars in the panel (Table 2). All these markers have exhibited high quality clustering as KASP assays when testing homozygous lines (Fig. 2). Of the six predictive SNP markers, 2BS-5194460_kwm747, 2BS-5192454_kwm677, 2BS-5175914_kwm847, and 2BS-5175914_kwm849 were best for distinguishing heterozygotes (Additional file 2: Figure S1) and are well suited for MAS of *Lr16* in wheat breeding programs.

**Table 2:** Haplotype data of wheat lines with six SNP loci co-segregating with *Lr16*

Wheat line or cultivar	IT^a^	*Lr16* status	BS00108724_kwm461	2BS-5192454_kwm677	2BS-5203447_kwm742	2BS-5194460_kwm747	2BS-5175914_kwm847	2BS-5175914_kwm849
McKenzie, RL4137	;1-	Carrier	a	a	a	a	a	a
AC Barrie, AC Domain, AC Majestic, Buck Manantial, Kenyon, RL6005 (Tc-Lr16)	1-	Carrier	a	a	a	a	a	a
BW278, Kanata, Prodigy	1+	Carrier	a	a	a	a	a	a
86ISMN 2137, 98B26-N1C01, AC Cadillac, Alikat, BW314a, CDC Teal, Frontana, McNeal, Neepawa, Park, Roblin, Sunstate	3-	Non-carrier	b	b	b	b	b	b
96B42-E3C, AAC Chiffon, AAC Indus, AC Meena, BW711, CDC Origin, CDC Thrive, Glenlea, Lancer, Lee, LMPG-6S, Marquis, RL6071, Sadash	3	Non-carrier	b	b	b	b	b	b
93464, 8802-EB3C, 94B30-C6D, 9606-AQ01C, 97B03-N2C, 98B25-AS5C03, 98B60-W1A, AC Abbey, AC Bellatrix, AC Crystal, AC Eatonia, AC Foremost, AC Michael, AC Nanda, AC Reed, AC Vista, Alvena, Apex, Ashby, Bhishaj, Biggar, Broatch's Whitehead, BW270, BW553, BW591, BW608, BW621, BW666, BW710, BW717, Canthatch, Canuck, Canus, CDC Bounty, CDC Harrier, CDC Kernen, CDC Makwa, CDC Nexon, CDC Osprey, CDC Ptarmigan, CDC Silex, CDN Bison, Chablis, Chinese Spring, Chinook, Conquer VB, Conway, Coronation, Cypress, Garnet, Hartog, Hoffman hrf, Huron, HY476, Katepwa, Kota, L8509-N5A, Ladoga, Lake, Leader, Manitou, Milan 13, Nandu, ND2827, Pacific, Percy, Pioneer-2375, Prelude, Preston, Radiant, Red Bobs 222, Red Fife, Redman, Regent, Reliance, Renfrew, Renown, Rescue, Reward, RL4452, Ruby, Saunders, SC8021-V2, Sinton, Snowhite476, Stanley, Supreme, Thatcher, W95132, White Fife	3+	Non-carrier	b	b	b	b	b	b
CL55, CL56, CL133, CP2911, Little Club	3 + 4	Non-carrier	b	b	b	b	b	b
Morocco	4	Non-carrier	b	b	b	b	b	b

^a^Infection type; resistant = ‘0’, ‘;’, ‘1’, or ‘2’, susceptible = ‘3’ or ‘4’

## Discussion

In the present work, we were able to develop numerous new markers that delineate the *Lr16* interval on the distal end of the short arm of chromosome 2B. A higher resolution SNP linkage map for *Lr16* was developed using four independent mapping populations, which represent approximately 768 (BW278/‘AC Foremost’, 384 RILs), approximately 188 (‘Kenyon’/86ISMN 2137, 94 RILs), 400 (‘AC Majestic’/‘Glenlea’, 400 DHs) and 172 (RL4452/‘AC Domain’, 172 DHs) gametes (Fig. 1). In total, 43 SNP and two PCR markers were mapped in the *Lr16* interval that was localized within ~ 1.8 cM.

*Lr16* is a race-specific seedling stage resistance gene [14]. Previously characterized seedling stage leaf rust resistance genes (e.g., *Lr1*, *Lr10* and *Lr21*) were found to be RGAs [9–11]. The majority of wheat R genes have coiled coil (CC), nucleotide-binding-site (NBS), and leucine-rich-repeat (LRR) motifs, and are referred to as CNL type R genes. Various studies have reported that R genes are often present in clusters in the genomes of many species. Here, we identified three RGA-like genes clustered near the *Lr16* locus and five SNP markers (BS00108724_kwm461, 2BS-5203447_kwm742, 2BS-5194460_kwm747, 2BS-5175914_kwm847, and 2BS-5175914_kwm849), which were developed from these RGAs and co-segregated with *Lr16* in all mapping populations (Fig. 1).

Stacking (or pyramiding) of multiple resistance genes is necessary to prolong resistance durability and enhance effective use of *Lr* genes [2]. *Lr16* has been shown to confer effective resistance in leaf rust nurseries when combined with adult-plant R genes such as *Lr34* and *Lr13* [16, 18, 41]. In fact, *Lr16* does not provide effective resistance in Canada when used as the sole source of leaf rust resistance [42]. Thus, diagnostic molecular markers are necessary to stack *Lr16* with other *Lr* genes through MAS. Here we found six SNP markers that were predictive of the presence or absence of *Lr16* in the diverse set of wheat germplasm tested (Table 2 and Additional file 1: Table S2). KASP assays were designed for SNPs that can readily be used in wheat breeding programs. The utility of the SNP markers was confirmed using a panel of 133 wheat lines/cultivars. The panel included reference stocks, spelt wheat, and a diverse set of cultivars and breeding lines from Canada, USA, Asia, Australia, North Africa, and South America with known genotype at the *Lr16* locus (Additional file 1: Table S2). The Canadian wheat cultivars and breeding lines comprised different marketing classes with different end-use functionality.

*Lr16* is believed to be originally sourced from five wheat cultivars, ‘Columbus’, ‘Warden’, ‘Exchange’, ‘Selkirk’, and ‘Etoile de Choisy’ [36]. ‘AC Domain’, ‘Kanata’ and ‘Columbus’ inherited *Lr16* from RL4137. Subsequently, the wheat line BW278 inherited *Lr16* from ‘AC Domain’, while ‘AC Barrie’, ‘AC Majestic’, ‘McKenzie’ and ‘Prodigy’ inherited *Lr16* from ‘Columbus’. For Kenyon, the source of *Lr16* was ‘Buck Manantial’. The wheat cultivar ‘Buck Manantial’ was originally released in Argentina and its source of *Lr16* could not be determined by the authors. The Thatcher-*Lr16* differential line RL6005 inherited *Lr16* from ‘Exchange’. Interestingly, both Kenyon and RL6005 have a different allele for the SSR marker wmc764 than the other *Lr16* carriers in this study [14]. The polymorphism at wmc764 between the *Lr16* carriers is most likely a relatively recent mutation since SNPs in the *Lr16* region do not differentiate the different sources of *Lr16*. Six SNP markers (BS00108724_kwm461, 2BS-5192454_kwm677, 2BS-5203447_kwm742, 2BS-5194460_kwm747, 2BS-5175914_kwm847, and 2BS-5175914_kwm849) presented in this work were completely predictive of *Lr16* in a diverse set of wheat germplasm and will be useful for MAS of *Lr16*. Interestingly, all the markers except 2BS-5192454_kwm677 were derived from RGAs (Additional file 1: Table S1). These new SNP markers will be useful in fine mapping and cloning of *Lr16*.

## Conclusions

Leaf rust resistance is a high priority for wheat breeders across the world. *Lr16* is a widely deployed R gene that provides effective resistance when pyramided with *Lr34. Lr16* was mapped to chromosome arm 2BS relative to numerous SNP markers in four mapping populations. The position of *Lr16* relative to the DNA markers was consistent in all crosses. Six SNPs co-segregating with *Lr16* were identified that are individually predictive for the presence/absence of *Lr16* in a diverse set of 133 wheat lines. In this study, several approaches were used to develop markers, including (1) using previously mapped SSR, DArT and SNP markers, (2) developing conserved primers using wEST, (3) mining SNP markers based on sequences of previously mapped markers or comparative synteny analysis with *Brachypodium* and rice, and (4) RGA-based SNP marker development. Of the 229 SNPs identified in this study, only 16 SNPs (7%) were developed from RGAs. However, five of the six predictive and co-segregating SNP markers were derived from RGAs. This demonstrates that RGA-based marker development is an effective approach for fine mapping and further cloning of resistance genes. The new predictive SNP markers developed here will enable efficient selection of *Lr16* in wheat breeding programs and will be useful for further cloning efforts of *Lr16*.

## Additional files

Additional file 1: Table S1. PCR primers for DNA markers used in the study. **Table S2.** *Lr16* haplotype panel sorted by *Lr16* status, country of origin, marketing class, and cultivar/line name. (XLSX 39 kb)
Additional file 2: Figure S1. Genotyping profile of the KASP markers (A) 2BS-5194460_kwm747, (B) 2BS-5192454_kwm677, (C) 2BS-5175914_kwm847, (D) 2BS-5175914_kwm849, (E) 2BS-5203447_kwm742, and (F) BS00108724_kwm461 tested on homozygous *Lr16* carriers, homozygous susceptible wheats, and heterozygous plants to show the diagnostic potential of the KASP assays to distinguish between heterozygous and homozygous samples. (PPTX 826 kb)

## Abbreviations

BSA: Bulked segregant analysis
CC-NB-LRR: Coiled-coil, nucleotide binding site, leucine rich repeat
CNL: Coiled-coil, nucleotide binding site, leucine rich repeat
CWRS: Canada western red spring
DArT: Diversity arrays technology
DH: Doubled haploid
KASP: Kompetitive allele specific PCR
MAS: Marker-assisted selection
R: Resistance
RGA: Resistance gene analog
RIL: Recombinant inbred line
SNP: Single nucleotide polymorphism
SSR: Simple sequence repeat
WEC: Whole exome capture
wEST: Wheat expressed sequence tag
