# Supplementary material for: Highly predictive SNP markers for efficient selection of the wheat leaf rust resistance gene Lr16
Source: BMC Plant Biol. 2017 Feb 15;17:45. doi: 10.1186/s12870-017-0993-7 (PMC5311853; doi:10.1186/s12870-017-0993-7)
Supplement: Additional file 2: Figure S1. — Genotyping profile of the KASP markers (A) 2BS-5194460_kwm747, (B) 2BS-5192454_kwm677, (C) 2BS-5175914_kwm847, (D) 2BS-5175914_kwm849, (E) 2BS-5203447_kwm742, and (F) BS00108724_kwm461 tested on homozygous Lr16 carriers, homozygous susceptible wheats, and heterozygous plants to show the diagnostic potential of the KASP assays to distinguish between heterozygous and homozygous samples. (PPTX 826 kb) [file 12870_2017_993_MOESM2_ESM.pptx]

## Slide 1
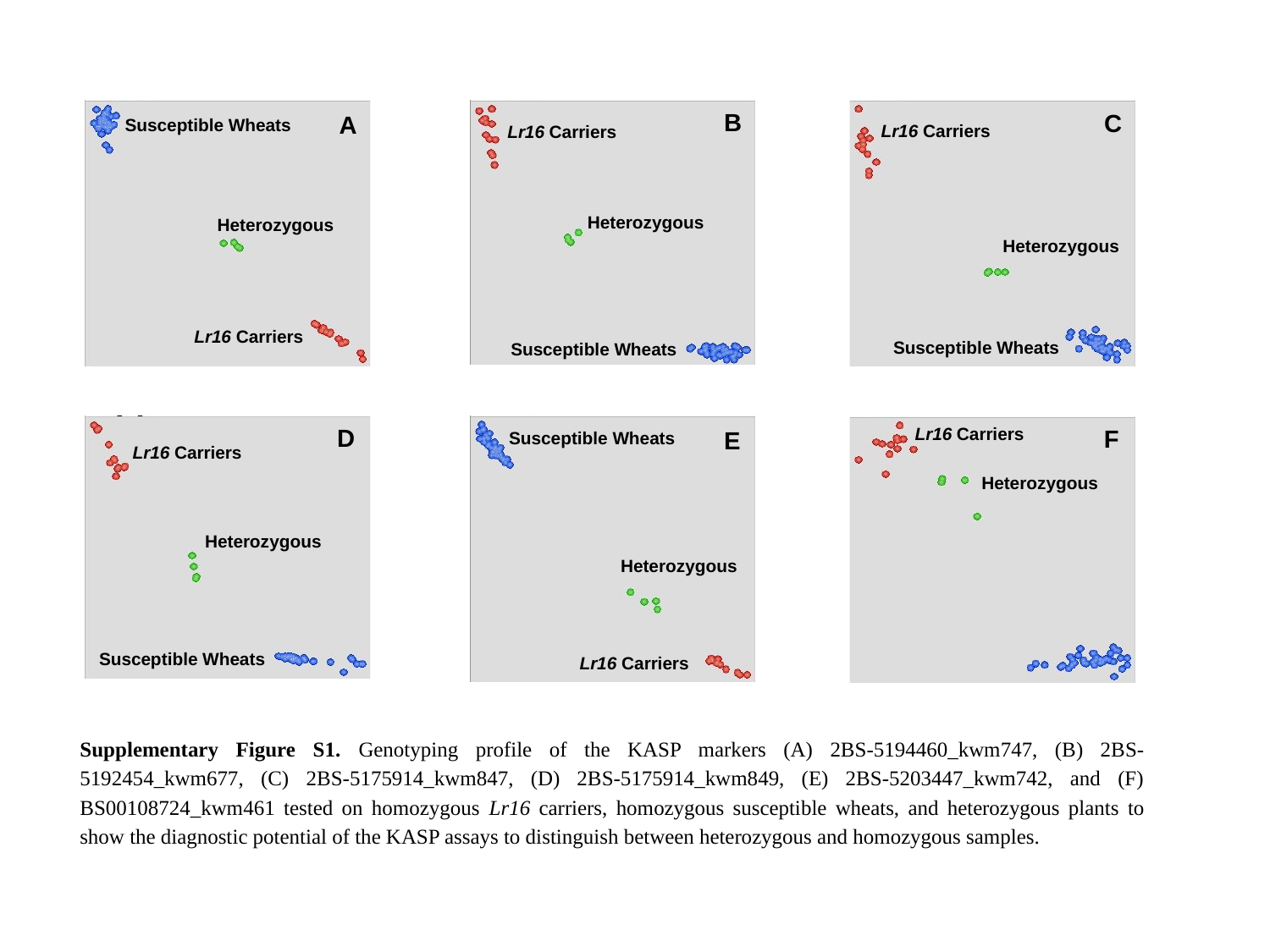

A
Susceptible Wheats
Heterozygous
Lr16 Carriers
B
Lr16 Carriers
Heterozygous
Susceptible Wheats
C
Lr16 Carriers
Heterozygous
Susceptible Wheats
D
Lr16 Carriers
Heterozygous
Susceptible Wheats
E
Susceptible Wheats
Heterozygous
Lr16 Carriers
Lr16 Carriers
F
Heterozygous
Susceptible Wheats
Supplementary Figure S1. Genotyping profile of the KASP markers (A) 2BS-5194460_kwm747, (B) 2BS-5192454_kwm677, (C) 2BS-5175914_kwm847, (D) 2BS-5175914_kwm849, (E) 2BS-5203447_kwm742, and (F) BS00108724_kwm461 tested on homozygous Lr16 carriers, homozygous susceptible wheats, and heterozygous plants to show the diagnostic potential of the KASP assays to distinguish between heterozygous and homozygous samples.
